# Preparation and Application of Sulfamethoxazole-Imprinted Polymer on Solid-Phase Extraction of Pharmaceuticals from Water

**DOI:** 10.3390/polym17233203

**Published:** 2025-11-30

**Authors:** Kristina Tolić Čop, Stjepan Jozinović, David Visentin, Dejan Milenković, Petra Vukovinski, Ramona Petko, Robert Vianello, Dragana Mutavdžić Pavlović

**Affiliations:** 1 Department of Analytical Chemistry, Faculty of Chemical Engineering and Technology, University of Zagreb, Trg Marka Marulića 19, 10000 Zagreb, Croatia; ktolic@fkit.unizg.hr (K.T.Č.);; 2 Department of Molecular and Systemic Biomedicine, Faculty of Biotechnology and Drug Development, University of Rijeka, Ul. Radmile Matejčić 2, 51000 Rijeka, Croatia; david.visentin@biotech.uniri.hr; 3 Department of Science Institute for Information Technologies, University of Kragujevac, Jovana Cvijića bb, 34000 Kragujevac, Serbia; dejanm@uni.kg.ac.rs; 4 Laboratory for the Computational Design and Synthesis of Functional Materials, Ruđer Bošković Institute, Bijenička Cesta 54, 10000 Zagreb, Croatia; robert.vianello@irb.hr

**Keywords:** pharmaceuticals, solid-phase extraction, molecularly imprinted polymer, wastewater, molecular dynamics simulation

## Abstract

Pharmaceutical compounds are small, invisible, and biologically powerful molecules that, due to widespread production and consumption, have become part of the environment, causing long-term adverse effects on biota even at low doses. Advances in sensitive and reliable analytical methods have made their detection possible in complex environmental matrices such as wastewater. Given the large number of synthesized pharmaceuticals with various therapeutic purposes, the occurrence of a synergistic effect is to be expected, interfering with their analysis. Therefore, the challenging analysis is often improved through the application of different sample preparation techniques. This paper includes the development of an SPE-HPLC-DAD method for the determination of eleven pharmaceuticals from water samples. To achieve better recoveries for the specified pharmaceutical (sulfamethoxazole) and possibly other components of the mixture, a sulfamethoxazole-imprinted polymer (MIP-SMETOX) was prepared and used in combination with a commercial sorbent (Oasis HLB) for MIP-SPE-HPLC-DAD. After optimization of the extraction conditions, both methods were validated. The LOD was 0.1 to 0.5 µg/L for SPE-HPLC-DAD and 0.1 to 0.25 µg/L for MIP-SPE-HPLC-DAD, depending on the pharmaceuticals. The matrix effect is different (77–196%) for both methods. A decrease in the signal for sulfamethoxazole (77%) was observed with SPE-HPLC-DAD, while MIP-SMETOX as a sorbent is not suitable for procaine (196%), and this is also the highest matrix effect. To extend the data obtained, additional in silico methods were used to gain deeper insights into the nature and strength of the binding interactions. Both methods (with and without MIP) confirmed their purpose by determining various validation performance features, and the final goal of the developed methods was tested using complex wastewater. The MIP-SMETOX produced justified its production, as the MIP-SPE-HPLC-DAD method is generally slightly better than the method using only a commercial sorbent.

## 1. Introduction

In the absence of restrictive regulations on small organic molecules such as pharmaceuticals, water pollution has become a global problem. For many decades, and even today, a wide range of new pollutants are continuously discharged into the environment from various industries, agriculture, wastewater treatment plants, improper disposal, households, etc. Numerous studies have shown that many pharmaceutical substances are not effectively removed during wastewater treatment and are not biodegradable in nature [1]. The incomplete removal of these compounds by wastewater treatment plants is considered to be one of the main sources of their release into the environment [2,3,4,5,6]. Once present in the environment, they can cause short-term and long-term negative effects on living organisms [7,8,9]. Although the existence of antibiotics has improved the quality of human and animal life, the widespread use of antibiotics has made them the most abundant therapeutic agents in the aquatic environment [9,10]. Sulfamethoxazole, a sulfonamide antibiotic, stands out due to its wide use in the prevention and treatment of broad-spectrum bacterial infections, and for this reason, is frequently detected in various water matrices. As it is characterized as a persistent, poorly biodegradable compound that has a strong impact on treatment resistance, it is important to develop various technologies to remove sulfamethoxazole and other harmful pharmaceuticals [11,12,13,14]. Sulfamethoxazole is generally present in the environment in the ng/L range; for example, Wu et al., 2015, and Shi et al., 2020, reported concentrations between 6.4 and 8488 ng/L [15,16,17].

It follows from all this that it is extremely important to monitor the movement of pharmaceuticals in the environment and, in particular, to determine their concentrations, which is possible by using highly sensitive analytical techniques. The simultaneous analysis of different groups of compounds, which often have different physico-chemical properties, poses a challenge for the optimization of experimental conditions. This complexity can lead to suboptimal performance in the detection of certain substances. Nevertheless, the development of multitarget methods is beneficial, as they facilitate routine analysis and generate extensive datasets [6]. Various chromatographic methods have been used for the analysis of pharmaceuticals in environmental matrices [18,19,20,21,22]. For the accurate and reliable analysis of trace analytes, the modernization of analytical techniques enabled the daily monitoring of pollutants at low concentrations in complicated matrices, which also provided us with important information on the fate and behavior of pharmaceuticals in the environment [23].

When it comes to sample preparation, solid-phase extraction (SPE) is considered the most popular, simple, and cost-effective technique for the pretreatment of various pollutants in different environmental samples with low use of organic solvents [24,25]. For the pollutant removal or purification of pollutants, it is important to use sorbents that have high selectivity for the target compounds. Although many commercial SPE sorbents are available on the market, their selectivity is often questionable due to the more complex environmental samples. New materials are needed to improve the efficiency of analyzing problematic compounds. One promising technique that supports the selective analysis of pollutants is the production of polymers imprinted with the molecule of interest. Molecularly imprinted polymers (MIPs) are a class of synthetic materials characterized by their ability to selectively recognize specific target molecules. The unique recognition properties of MIPs result from a template-assisted polymerization process that creates a molecular memory in the polymer matrix [26]. There are different ways of synthesizing molecularly imprinted polymers (MIPs), such as non-covalent and covalent polymerization and conventional bulk, in situ, precipitation, emulsion, suspension, surface imprinting, and sol–gel polymerization. The general process involves the reaction between the template molecule and a functional monomer unit (methacrylic acid (MAA), 4-vinylpyridine (4-VP), acrylamide, methacrylamide, 2-hydroxyethyl methacrylate (2-HEMA), etc.) with the addition of cross-linker (*p*-divinylbenzene (DVB), ethylene glycol dimethacrylate (EGDMA), trimethylpropane trimethacrylate (TRIM), etc.) and initiator (azobisisobutyronitrile (AIBN), azobisdimethylvaleronitrile (ABDV), benzoyl peroxide (BPO), etc.) in a suitable porogen and under the influence of elevated temperature or UV light. After washing the template from the polymer, the result of the polymerization is a specific size, pore shape, and functional groups that allow the uptake of the target pharmaceutical [25,27,28,29,30,31]. This type of highly selective sorbent has been widely used for sorption and extraction experiments for various types of pharmaceuticals, such as atenolol [28], fluconazole [24], the antibiotics ciprofloxacin, norfloxacin, tylosin, and ofloxacin [31,32], the non-steroidal anti-inflammatory drug diclofenac and ketoprofen [31], and many more. A traditional approach, such as the bulk polymerization described above, remains one of the most widely used synthesis methods. Although this method is time consuming, as polymerization takes hours, the MIP preparation itself is characterized as robust, simple, and low cost, requiring minimal laboratory equipment. It produces mechanically and thermally stable materials with excellent reproducibility and flexibility in using different monomers and template molecules. Additionally, polymer purification is minimal, with few residues. Sometimes, issues such as particle grinding and non-specific interactions that form heterogeneous cavities are addressed by immobilizing different substrates (e.g., sol-gel imprinting, electropolymerization, surface imprinting), such as magnetic materials, which promote sensitivity [29,33]. Although MIPs are primarily synthesized to remove the template molecule based on the “key-lock” principle [34], their performance can sometimes extend to other molecules. Non-specific interactions may occur between the formed cavities and other target molecules that are structurally similar to the original template (size, shape, functional groups) [27,35,36]. For example, Caro et al. prepared a polymer imprinted with the molecule of ibuprofen, which was successfully applied for the solid-phase extraction of other anti-inflammatory drugs such as benzoic acid, naproxen, diclofenac, and fenoprofren [37]. Also, there are studies where MIP with a single molecular imprint is used for the extraction of pharmaceuticals from several different therapeutic groups, achieving more sensitive results for other components from the matrix [38].

Considering the described concern about the existence of small, invisible, and toxic organic molecules in water, an optimized SPE method for the analysis of twelve pharmaceuticals with different physicochemical properties was developed, validated, and finally applied to wastewater samples in this work. Taking into account the complexity of the water matrix and the simplicity of the preparation of the selective sorbent, the MIP was further prepared by bulk polymerization for the selective recognition of the sulfamethoxazole molecule to achieve a better recovery of this and possibly some other molecules. The efficacy of the prepared sorbent was tested on the same mixture of pharmaceuticals using the same optimized SPE method without addition and with the addition of a commercial HLB sorbent. The analysis was supported by high-performance liquid chromatography coupled with a diode array detector. Both developed methods were validated and applied to real wastewater samples. To support this process, in silico methods were employed to simulate the post-polymerization stage intermolecular interactions occurring between the polymer and the target pharmaceuticals. In recent years, MIP preparation has transitioned from a randomized trial-and-error process to a more rational approach using computational methods [39]. These techniques have garnered significant traction as they are seen as a faster, cheaper, and more environmentally friendly alternative for MIP design and preparation. Commonly involved approaches include molecular mechanics (MM), molecular dynamics (MD), and quantum mechanics (QM) [39,40]. QM methods provide highly accurate data but require substantial computational resources to run them, limiting their widespread application [41]. For that reason, their use commonly employs hybrid methods combining QM, MD, and MM, balancing accuracy and efficiency [39,41]. Machine learning has also emerged as a promising tool for interaction prediction, but is constrained by the extensive datasets needed to train accurate models that are optimized for a wide range of polymer-pharmaceutical combinations [42]. Recent examples of using these methods include the design of an MIP for filtering citrinin and related toxins [43]. Also, it is demonstrated that computational methods provide valid predictions for predicting experimental results where the methods correctly predicted atropine and hyoscine affinity for the following monomers: acrylic acid (AAC), methacrylic acid (MAA), and acrylamide (ACR) [44].

## 2. Materials and Methods

### 2.1. Pharmaceutical Standards, Chemicals, and Materials

The tested mixture consisted of twelve pharmaceuticals with high purity grade (≥98%): (amoxicillin (AMX), atenolol (ATL), dexamethasone (DEXA), diazepam (DIA), diclofenac (DCF), carbamazepine (CBZ), ofloxacine (OFX), procaine (PRO), sulfamethazine (SMETH), sulfamethoxazole (SMETOX), torasemide (TOR), *β*-estradiol (E2)); their physicochemical properties and suppliers are listed in Table S1. All solvents used: methanol (Fisher Scientific, Waltham, MA, USA), ethanol (Honeywell, Charlotte, NC, USA), acetonitrile (Carlo Erba, Milan, Italy), acetone (Gram-Mol, Zagreb, Croatia), ethyl acetate (T.T.T. d.o.o., Novaki, Croatia), 1-propanol and 2-propanol (Fischer Chemicals, Waltham, MA, USA), dichloromethane (Kemika, Zagreb, Croatia), hexane (Kemika, Zagreb, Croatia), toluene (Kemika, Zagreb, Croatia), diethyl ether (Kemika, Zagreb, Croatia) were pro-analysis or HPLC grade as purity grade. Ultrapure water was prepared using a Milipore Milli-Q Plus water purification system (Millipore, Paris, France). The stock standard solution of pharmaceuticals was prepared in an adequate solvent (Milli-Q water, methanol, or acetonitrile) by accurate weighing of each powder separately to obtain a final concentration of 1000 mg/L. The working standard solutions were prepared for each experiment by mixing and diluting the stock solutions with methanol. All prepared standard solutions were stored in the dark at 4 °C. Chemicals used for pH adjusting were hydrochloric acid solution (Gram-mol, Zagreb, Croatia) and sodium hydroxide (VWR Chemicals, Radnor, PA, USA) (0.1 M). Chemicals for the polymer syntheses, methacrylic acid (MAA) and ethylene glycol dimethacrylate (EGDMA), were purchased from TCI (Tokyo, Japan). Futhermore, 2-hydroxyethyl methacrylate (HEMA) was supplied by Acros Organics (Antwerpen, Belgium), and the initiator 2,2′-azobis-isobutyronitrile (AIBN) was supplied by Sigma Aldrich (St. Louis, MO, USA).

For solid-phase extraction, the Oasis HLB 60 mg/3 mL cartridge from Waters (Milford, MA, USA) was used due to its wide spectrum of applicability with high recoveries for non-polar to polar compounds. The polypropylene SPE empty reservoir (3 mL) and adequate 20 µm polyethylene frits (PTFE) were purchased from Agilent (Santa Clara, CA, USA) with the aim of preparing MIP-HLB sorbent in one SPE column.

### 2.2. Preparation of Molecular Imprinted Polymers

The template molecule (SMETOX, 0.25 mmol) was dissolved in acetonitrile as a porogen and placed in a glass tube. Then, the functional monomer (MAA, 1.0 mmol or HEMA, 1.0 mmol), the crosslinking monomer (EGDMA, 5.0 mmol), and the initiator of radical polymerization (AIBN, 0.12 mmol) were added. Three different MIPs were synthesized, varying the amount of porogen—the volume of acetonitrile 3, 6, and 12 mL was tested. The polymerization mixture was shaken in a Vortex shaker and degassed with a stream of N_2_ for 5 min to remove oxygen traces. A non-imprinted polymer (NIP) containing no template was also prepared using the same procedure. The tube was sealed and left in the dryer at 60 °C for 24 h. After polymerization time, the tube was carefully shattered, the solid polymer was removed from the glass and ground. The template molecule was extracted by washing the obtained MIP with a mixture of methanol:acetic acid (9:1) (*v*/*v*), followed by pure methanol. Washing was carried out until the absence of the chromatographic curve of SMETOX, as well as its specific absorption spectrum, was detected in the chromatogram. This was sufficient for the next phase of the experiment, i.e., the printed areas in the prepared MIP-SMETOX were ready for contact with the sulfamethoxazole-containing water sample. The MIP particles were finally dried before using.

### 2.3. Structural Characterization of Imprinted Polymers

The characterization of MIP and NIP composites was investigated by SEM analysis (TESCAN Vega III Easyprobe) to observe the morphology of the prepared materials. The infrared spectra of the investigated materials were analyzed by an FTIR spectrometer (Vertex 70v, Bruker, Billerica, MA, USA) equipped with an attenuated total reflection (ATR) accessory with a diamond crystal. Sixteen scans were collected for each measurement over the spectral range of 400–4000 cm^−1^ with a resolution of 4 cm^−1^. By FTIR comparison, analyses of MIP powder before and after washout, with pure SMETOX standard, were observed. The aim of this comparison is to determine the part of the SMETOX spectrum that should be found in unwashed MIPs, and its absence in washed MIPs, which should also be a confirmation of a successful washing process.

### 2.4. Water Sample Preparation

In the development and optimization of the method, real water without pharmaceutical additives was used. The water was taken from the Mikulići Spring, Zagreb, Croatia. Immediately before analysis, water samples were filtered through 0.45 μm nylon filters (Pall Corporation, Washington, NY, USA) to remove particles. The optimized and validated SPE-DAD method was applied to real wastewater sample analysis. For the application of the developed extraction method, real hospital wastewater sampled in the Italian region of Emilia-Romagna was used [2]. Immediately prior to the analysis of said water, the water sample was thawed, whereupon the macroparticles were first removed by vacuum filtration through a Büchner funnel using the “blue band filter”, which has the smallest pores, and the “white band filter”. After coarse filtration, the wastewater is filtered by vacuum filtration using PTFE membrane filters (47 mm, 0.45 µm). The filtered water was stored in a refrigerator at a temperature of 4 °C until the experiments were carried out.

### 2.5. Solid-Phase Extraction—Method Development, Validation, and Application on Real Samples

The SPE procedure involves a few steps: conditioning the Oasis HLB cartridges with 2 mL of methanol (the sorbent must not be dry); retention of the analytes (1 mL) in 100 mL of spring water with an adjusted pH at a constant flow rate of 4 mL/min (the cartridge is dried with vacuum until the water is removed); elution of the retained analytes with 5 × 2 mL of solvent; evaporation to dryness at 40 °C on a rotary evaporator; and finally dissolution of the dry residue in 1 mL of methanol. Each experiment was performed in triplicate and with a blank sample (water without pharmaceuticals). In the manner described, sets of experiments were carried out with the aim of determining the parameters that would give the best recovery values for the pharmaceutical mixture during the extraction process. The first set of experiments involved testing the pH value of spring water, where the water sample was adjusted to pH 3, 4, 5, 6, 7, 8, and 9 by adding 0.1 M hydrochloric acid or sodium hydroxide. After selecting the optimum pH value, elution of the pharmaceuticals was carried out with organic solvents such as methanol, ethanol, isopropanol, ethyl-acetate, acetonitrile, and acetone. The optimal recovery rates for the entire mixture were obtained with methanol, so that the elution volumes were changed in the next experiments (1 × 2 mL, 2 × 2 mL, 3 × 2 mL, 4 × 2 mL, 5 × 2 mL, 6 × 2 mL). In the final experiment, the sample volume was tested, with extractions performed in 50, 100, 250, and 500 mL of water.

The SPE-HPLC-DAD method was then validated under optimal conditions by evaluating the following performance characteristics: linearity range, LOD, and LOQ when performing the extraction test in the concentration range of 0.01–5 mg/L; precision as repeatability and reproducibility in nine replicates by spiking water with a standard solution of 1 mg/L within a time interval of two days; the matrix effect by comparing the area for each pharmaceutical after extraction spike and their area in the standard solution (0.5 mg/L, 1 mg/L, 2 mg/L, and 5 mg/L). Finally, the developed and validated method was applied to real samples. Taking into account the wastewater load, the standard addition calibration method for the analysis of pharmaceuticals was applied. The extraction was performed three times for each sample, once without and twice with the addition of the standard solutions (0.5 and 1 mg/L).

The optimal parameters of the SPE-HPLC-DAD method were transferred to the MIP-SPE-HPLC-DAD method, with the exception that in the MIP-SPE-HPLC-DAD method, the influence of the order of serial connection of the prepared MIP with the commercial Oasis-HLB sorbent previously used in SPE-HPLC-DAD was investigated. In the serial connection of the prepared sulfamethoxazole-imprinted sorbent and the commercial Oasis HLB sorbent, the cartridges were packed in two different ways. In the first series, a frit was placed into the cartridge, followed by 60 mg of MIP-SMETOX, another frit, and then 60 mg of Oasis HLB sorbent. In the second configuration, the positions of the sorbents were reversed. The aim was to make the best possible extraction recovery, primarily for sulfamethoxazole, but also potentially for other tested pharmaceuticals in the mixture. After the optimization of the MIP-SPE-HPLC-DAD method, the validation and application of the method to the same sample of real water was started.

### 2.6. HPLC Analysis

A sample analysis was performed using an Agilent Series 1000 high-performance chromatograph coupled with a DAD detector (Santa Clara, CA, USA). Separation of analytes was achieved on Phenomenex C18 stationary phase (150 × 4.6 mm and particle size 5 µm) with elution with mobile phase consisting of 0.1% formic acid in Milli-Q water (eluent A) and acetonitrile (eluent B). The flow rate was adjusted to 0.5 mL/min with an injection volume of 30 µL. The gradient change in the composition of the mobile phase is shown in Table 1. The detection of analytes was monitored at 230, 240, 254, 275, and 290 nm, depending on the absorption spectra of each pharmaceutical. The purpose of the HPLC method was approved by the determination of validation performance characteristics such as linearity, limit of detection and quantification (LOD and LOQ), selectivity, sensitivity, accuracy, precision, and robustness.

The obtained chromatogram with associated retention time for each pharmaceutical is attached in Supplementary Materials (Figure S1).

### 2.7. Molecular Dynamics and System Preparation

The system was assembled and prepared using a custom in-house pipeline designed for molecular dynamics system setup. A single homo syndiotactic polymer of HLB and MAA, with 10 degrees of polymerization and both ends of the chain terminated with hydrogens, was modeled using molecular modeling software called Winmostar V11.8.2 [45]. In this paper, we have used a Generalized Amber Force Field (GAFF) [46] for modeling both polymers. GAFF is compatible with the AMBER force field, and it has parameters for almost all the organic molecules made of C, N, O, H, S, P, F, Cl, Br, and I. As a comprehensive force field, GAFF is suitable for the study of a great number of molecules (such as database searching) in an automatic fashion.

Ligand structures were provided in a CSV file format as SMILES strings. These strings were first standardized and cleaned, then converted to 3D structures using the RDKit package [47]. Adjusted protonation states for the desired pH were assigned using OpenBabel 3.1.0 [48], followed by energy minimization using the MMFF94 force field [49]. For pharmaceuticals with p*K*_a_ values close to the target pH, the built-in OpenBabel pH solver occasionally produced incorrect structures. For these cases, protonation states and charges were manually corrected and minimized using Avogadro 1.2.0 [50]. Subsequently, molecular parameters were generated with acpype [51] using the GAFF force field. Topologies were merged using ParmEd [52], and user-defined systems were constructed with PACKMOL [53], which ensures appropriate 3D organization of the molecules. The final systems were solvated in Simple Point Charge water (SPC) and neutralized using GROMACS 2023.1 [54,55]. Non-bonded interactions were treated using a Verlet cutoff scheme, with a cutoff of 1.2 nm for Coulomb and van der Waals interactions [56]. Long-range electrostatics were calculated using the PME algorithm [57]. Excluding production, all subsequent molecular dynamics simulations were conducted with constraints applied to hydrogen bonds using the LINCS algorithm [58].

The system was equilibrated through a four-step protocol: energy minimization using the steepest descent algorithm, followed by equilibration in the NVT and NPT ensembles. The initial pressure coupling was performed with the Berendsen barostat for stability, and the final equilibration employed the Parrinello–Rahman barostat to ensure proper structural relaxation over a total equilibration time of 1 ns [59,60]. Production simulations were run for 100 ns with a time step of 0.5 fs. Temperature was maintained at 293.15 K with the Nose–Hoover thermostat, and pressure was set to 1 atm with Parrinello–Rahman barostat [61].

All simulations were conducted using Supek, Croatia’s national supercomputer, which utilizes the PBSPro workload manager [62,63]. The molecular dynamics jobs were executed in parallel on CPU clusters with multithreading enabled set to run with 4 processes, each utilizing 4 threads, for a total of 16 threads per simulation.

Interaction energy calculations were performed using gmx_MMPBSA 1.6.2, employing the Poisson–Boltzmann (PB) approach for free energy estimation [64]. Input files for the analysis were generated using the “—create_input” command-line option provided by gmx_MMPBSA, with minimal modifications to the default settings. Adjustments included specifying the temperature, the simulation frame range, and incorporating the GAFF force field for parameter compatibility.

Pre-processing of the simulation trajectory was conducted to prepare the data for interaction energy calculations, following the guidelines recommended by gmx_MMPBSA. The custom group that included the polymer and pharmaceutical was defined in GROMACS using the “make_ndx” tool. The trajectory was then centered, and periodic boundary condition artifacts were removed using the newly created group as the target group. A subsequent step involved fitting the trajectory using a least-squares method (rot+trans option), to correct for rotational and translational movements. The processed and fitted trajectory was utilized as an input for interaction energy calculations.

## 3. Results

### 3.1. Optimization of SPE-HPLC-DAD Method

Sample preparation is one of the most important steps in the analysis. In solid-phase extraction, the choice of the appropriate sorbent with which to isolate the desired analytes is critical, as the choice of sorbent directly affects the performance of the method, i.e., its selectivity and capacity. This is primarily related to the physico-chemical properties of the analytes of interest, but the sample and its loading must also not be neglected, as well as understanding the interactions between the analyte and the sorbent in the environment of a complex matrix such as a real sample. Solid-phase extraction is the most commonly used preparation method for a wide range of water samples due to its simplicity and compatibility with analytes with different physicochemical characteristics [65]. The development of the SPE method included in the first step optimization of the pH value of the water sample due to its dependence on pharmaceuticals, i.e., induced stability, whether they are in protonated, neutral, or deprotonated form [66,67].

**Figure 1:**
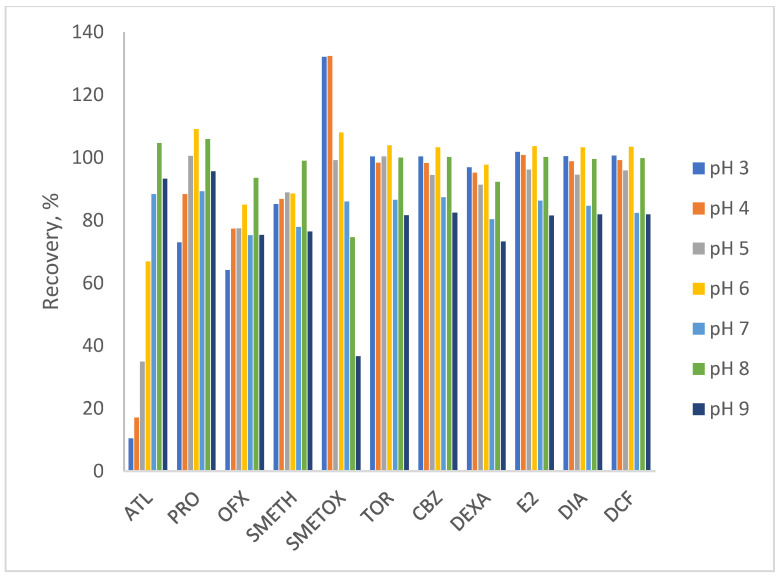
Testing the pH value of water for optimal extraction (pH range 3–9).

**Figure 2:**
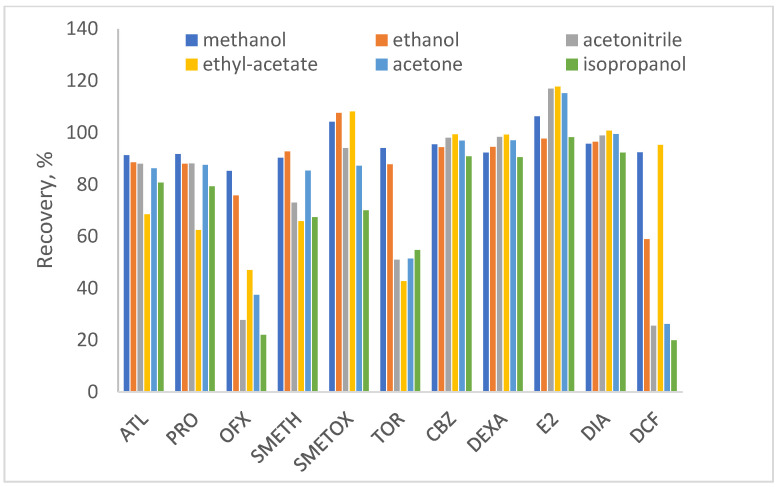
Organic solvent tested for optimal extraction (initial pH of water = 8, elution volume = 5 × 2 mL, sample volume = 100 mL).

When testing the pH range of the water from acidic to basic (Figure 1), the best performance for the extraction of the pharmaceutical mixture from the Oasis HLB sorbent was obtained at pH 8. As can be seen in Figure 1, all pharmaceuticals achieved recoveries of 92 to 105% with RSD values < 5.7%. SMETOX is the only analyte that achieves slightly lower recoveries in slightly alkaline pH (74%). It favors slightly acidic conditions, but during the development of the extraction method, generally optimal conditions are selected for the entire mixture, so, regardless of the somewhat lower recoveries for SMETOX itself, pH 8 is chosen as optimal. Through the next experiments, the selection of the solvent for elution was conducted. Solvents are chosen according to the criterion of the highest polarity index. It is determined separately for each sorbent and is defined by the eluotropic series of solvents. It enables reducing the consumption of the necessary solvent and increases the sorbent capacity. The choice of solvent is influenced by interactions between polar functional groups and sorbents, which change the solubility of the analyte [68]. According to the results obtained (Figure 2), the analytes were extracted best with protic solvent—methanol (recoveries > 90%), which is expected due to the possibility to form hydrogen bonds with the adsorbed water sample [69,70]. The use of other solvents, in particular aprotic solvents—acetonitrile, acetone, and especially ethyl acetate, led to a decrease in the recoveries of all pharmaceuticals tested.

**Figure 3:**
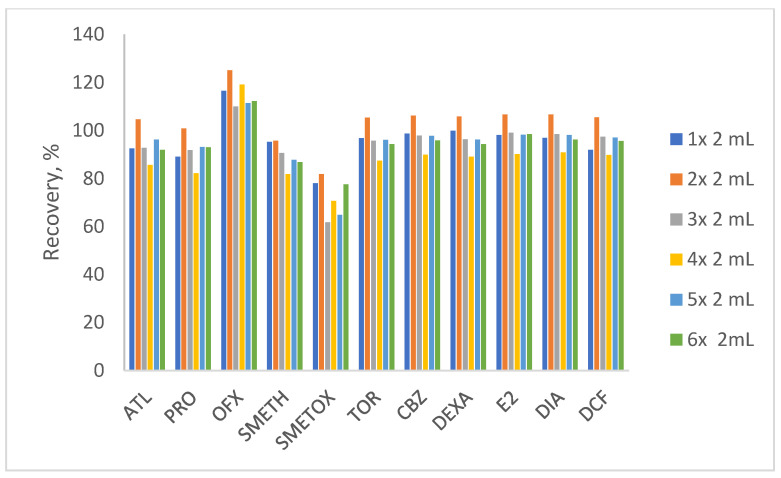
The methanol volume tested for optimal extraction (initial pH of water = 8, elution volume tested 2–12 mL, sample volume = 100 mL).

**Figure 4:**
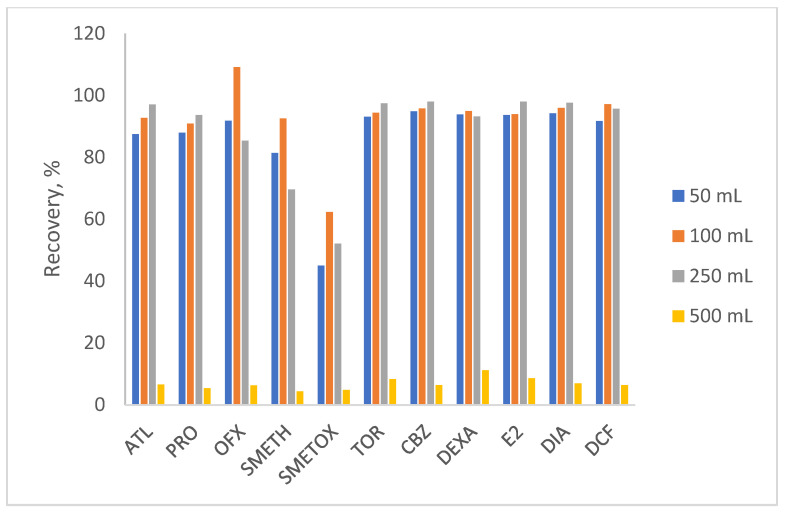
The sample volume tested for optimal extraction (initial water pH = 8, elution solvent = methanol, elution volume = 2 × 2 mL).

In accordance with the previous experiment, the aim of the next experiment was to reduce the volume consumption of the organic solvent without compromising the high recovery rates obtained with 5 × 2 mL methanol. Therefore, the elution was performed with the addition of 1 to 4 portions of 2 mL solvent. Furthermore, the extraction was additionally performed with a larger volume of eluent (6 × 2 mL). The results (Figure 3) showed the best elution with 2 × 2 mL of methanol, with recoveries of 95% to 107%, except for ofloxacin and sulfamethoxazole. The result obtained is extremely favorable due to the low consumption of organic solvent and the lower waste disposal costs. Ofloxacin at 125% indicates the matrix effect, while recoveries around 80% for SMETOX are logical due to the applied conditions. The last experiment to optimize the SPE method involved changing the sample volume (50–500 mL). The results obtained (Figure 4) showed satisfactory recoveries when using 100 and 250 mL of source water. However, 100 mL was chosen to minimize sample consumption, time of performance, and to avoid possible clogging of the sorbent and tubing, as it was the highest volume used.

### 3.2. Validation of SPE-HPLC-DAD Method

**Figure 5:**
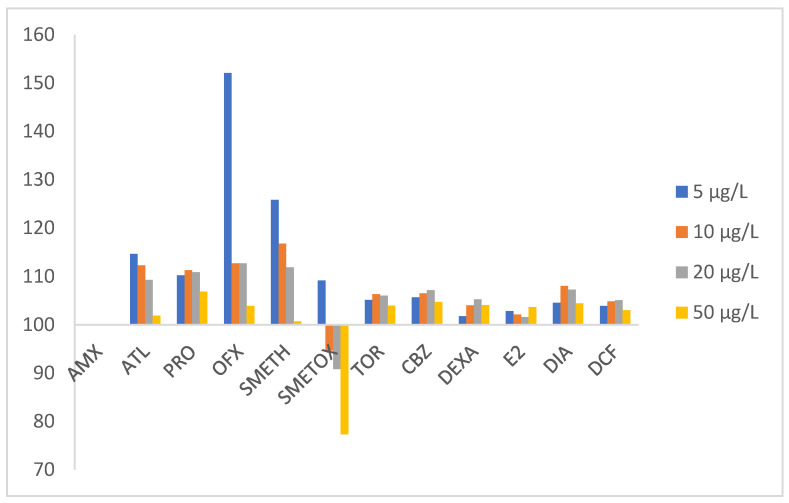
The matrix effect on SPE-HPLC-DAD method (initial pH of water = 8, elution solvent = methanol, elution volume = 2 × 2 mL, sample volume = 100 mL).

In the SPE method, the conditions were optimized as follows: a 100 mL spiked water sample eluted with 2 × 2 mL of methanol was validated by determining linearity, LOD, LOQ, precision, and trueness. The numerical values of the above validation characteristics are summarized in Table S2. Linearity, confirmed with correlation coefficients (R2) higher than 0.9774, was estimated for each analyte and ranged from 0.00025 to 0.001 mg/L. The LOD and LOQ were estimated experimentally using the signal-to-noise ratio (S/N) by comparing the signal of each analyte with the blank signal to achieve the ratio S/N = 3/1 for the LOD and 10/1 for the LOQ. Pharmaceuticals were extracted in the range of 0.00025 to 0.0005 mg/L for LOD and 0.00025 to 0.001 mg/L for LOQ, respectively. The precision as a relative standard deviation based on five replicates on the same day (repeatability) and over three days (reproducibility) was in a satisfactory range, below 9.39 and 14.29%, respectively. The influence of the matrix due to the complexity of environmental samples can cause difficulties in the detection of analytes, which has a negative impact on the accuracy and precision of the developed method [71,72]. The response signal may be suppressed or falsely greater than the actual value. The pharmaceutical signal in the source water was investigated by comparing it with the analyte response signal from standard solutions at concentrations of 0.5 mg/L, 1 mg/L, 2 mg/L, and 5 mg/L (corresponding to a concentration of 5, 10, 20, and 50 µg/L in water). The results obtained are summarized in Figure 5.

For most target compounds, a trend can be seen that the matrix effect decreases with increasing concentration. Moreover, the matrix effect is negligible for TOR, CBZ, DEXA, E2, DIA, and DCF, with recoveries in the range of 100 ± 10%, while the highest false-positive signal was observed for OFX and SMETH at the lowest concentration. The sample matrix caused a decrease in SMETOX recoveries at two lower concentrations, while the highest signal suppression was observed at 5 mg/L.

### 3.3. Preparation of Sulfamethoxazole-Imprinted Polymers

#### 3.3.1. Selection of a Functional Monomer

In many cases where a large number of analyte traces need to be extracted from real samples, it is difficult to meet the conditions for extracting all the desired analytes. In these cases, compromises have to be made between the extraction efficiencies achieved for the analytes analyzed, so that sometimes the efficiency of some analytes is reduced in order to increase the extraction efficiency of the more “problematic” analytes. In recent years, the preparation of polymeric sorbents with molecular imprints has been proposed as a solution to the above issue of the efficiency of the analytes of interest. Sulfamethoxazole is a drug that is widely used in both human and veterinary medicine. As it is less degradable and easily passes through water treatment plants, it is frequently found in surface water samples [73,74]. For this reason, it was not removed from the list of priority substances of the Water Framework Directive for many years under the leadership of the European Union [75,76]. This is also one of the reasons why this particular pharmaceutical was selected as a template molecule for the preparation of MIPs. In addition, sulfamethoxazole was selected for the preparation of the polymer sorbent because the efficiency values of its extraction from the mixture with 10 other pharmaceuticals are lower compared to the other pharmaceuticals in the mixture.

For the above reasons, the preparation of a specific sorbent with the imprint of the sulfamethoxazole molecule was started. For this purpose, two types of functional monomers (MAA and HEMA) were used, as described in Section 2.2, using an initial volume of 3 mL of acetonitrile as solvent. After removing the sulfamethoxazole as the template molecule and passing the sulfamethoxazole solution through the prepared sorbents with and without a template molecule, the results are shown in Table 2.

Based on the RSD values obtained, with an acceptance criterion of up to 10%, and the efficiency, which is preferably in the value range of 90–110%, a slightly higher efficiency of the MIP_MAA_ sorbent was found compared to the MIP_HEMA_ sorbent. This is due to the hydrophobic nature of HEMA and the hydrophilic nature of MAA. The results also show a higher binding efficiency of the sulfamethoxazole molecules on the surface of the MIPs compared to the NIP sorbents, which was assumed considering that the NIPs were synthesized without the presence of a template molecule. The results obtained follow the expected trend, but cannot be considered satisfactory due to the low efficiency values for any sorbent.

Regarding all the prepared polymers, the distribution coefficient *k* of SMETOX by MIPs and NIPs, as well as imprinting factors (*IF*s), were calculated by Equations (1) and (2) [77,78].

(1) $$k^{SMETOX}=\frac{\left(c_{0}-c\right)}{c}\times \frac{V}{m}$$

(2) $$IF=\frac{k_{MIP}^{SMETOX}}{k_{NIP}^{SMETOX}}$$

where *c*_o_ and *c* represent the initial and final concentrations, *V* represents the volume (mL) of the solution and *m* is the mass (g) of the tested polymer.

Based on these values, it was concluded that the MIP_MAA_ has a higher affinity for SMETOX than the corresponding NIP_MAA_ polymer and other polymers prepared with other functional monomers. The imprinting factors obtained (IF_MAA 1_ = 3.54; IF_HEMA_ = 2.14) for the MIP_MAA_ mentioned are an indication of a good imprinting effect. Comparing the IF obtained with that obtained in the previous paper [78], the results are as expected and again favor methacrylic acid, which has been shown to be a better functional monomer for the sulfonamide group of pharmaceuticals than 2-hydroxyethyl methacrylate, which was also tested. For this reason, all further experiments were carried out with methacrylic acid as the functional monomer, as slightly higher efficiency values were obtained for the molecule under investigation, sulfamethoxazole, and the use of sorbents with MAA as the functional monomer was found to be more effective in the literature [24,79]. The reason for this is that the sulfamethoxazole molecule is more suitable for the hydrophilic methacrylic acid molecule, probably due to its hydrophilic nature.

Geometrically optimized structures of MAA and sulfamethoxazole interact at two points via H-bonds with the MAA carboxylic acid with S=O and NH sulfonamide moieties or/and H-bonds with the MAA carboxylic acid with sulfonyl O atom and NH groups. This is due to the fact that MAA can act as both a hydrogen bond donor and acceptor and can therefore interact with the template at one or two interaction points and form MAA dimers. On the other hand, interaction with another functional monomer under study would lead to a more homogeneous binding site, as previously illustrated by the smaller number of possible interaction modes [78,80]. These observations indicate that the sulfamethoxazole-imprinted polymer likely has heterogeneous binding sites when interacting with MAA, which is the reason for the better imprinting effect and selectivity.

The following experiments on the preparation of the polymer sorbent with and without imprinting the sulfamethoxazole molecule were carried out with a larger volume of acetonitrile as solvent (6 and 12 mL) compared to the previous series of polymer sorbents (3 mL), since it was assumed that a larger amount of acetonitrile would create a more porous structure of the sorbent, which would allow a more selective and better binding of the template molecule as well as other molecules on its surface. The results of the experiments in which the sulfamethoxazole solution was passed through the previously washed sorbents are shown in Table 3.

The results presented in Table 3 show that the volume of solvent (6 mL for MIP_MAA 2_ and 12 mL for MIP_MAA 3_; 3 mL is the previous experiment in Table 2) is nevertheless crucial in the preparation of imprinted polymers, as significantly higher extraction efficiency was obtained for sulfamethoxazole-imprinted polymers, with the difference between identical MIPs and NIPs being significantly greater. The fact that a higher imprinting effect of sulfamethoxazole was obtained on the polymers produced is also confirmed by the IF values obtained (IF_MAA 2_ = 24.97 and IF_MAA 3_ = 149.83). All this suggests that all future experiments will be carried out with MIP_MAA 3_.

#### 3.3.2. Characterization of Prepared Polymers

The MIPs, as well as all the prepared NIPs, were analyzed by FTIR spectroscopy. Characterization of the FTIR device was carried out with the aim of determining the chemical structure of MIPs, NIPs, and washed MIPs from whose surface sulfamethoxazole was removed as a template.

It is noticeable that the spectra of all non-imprinted polymer samples overlap, which indicates a similar structure of the prepared polymer samples. Also, two intense vibrational bands are noticeable at wavenumbers of 1725 and 1145 cm^−1^. The vibrational band at 1725 cm^−1^ corresponds to the stretching of the C=O bond, while the vibrational band at 1145 cm^−1^ corresponds to the C-O-C bond stretching. Likewise, the weak vibrational stretches of the C=C vinyl group, which can be observed at 1631 cm^−1^, represent the conversion of the monomer into a polymer, or, in other words, indicate a successfully carried out polymerization. During the polymerization reactions by the free radical mechanism, the double bond of the vinyl group is converted into a single C-C bond, and therefore, a reduced intensity or absence of this band can be expected after polymerization. The bands in the fingerprint area, below 1000 cm^−1^, describe the molecules in more detail because, namely, it is possible to determine the stretching vibrations of C-C bonds (880 cm^−1^), out-of-plane deformations of C-H bonds (960 cm^−1^), and their twisting and swaying (814 cm^−1^). The only difference between the prepared non-imprinted polymers is that in the NIP-HEMA spectrum, a broad band of low intensity is found at 3500 cm^−1^, which represents the stretching of the O-H bond of the hydroxyl group. The inability to observe (or a weaker possibility of) this band may be a consequence of the poor purity of the prepared polymer or unsuccessful polymerization. Based on the preliminary results (Table 3) as well as this observation, further experiments were focused exclusively on methacrylic acid as a functional monomer for the preparation of MIPs-SMETOX.

Infrared spectra confirmed the presence of SMETOX in all the MIP-polymer sorbents. Spectra of sulfamethoxazole-imprinted polymers show an increase in the intensity of all previously mentioned bands due to the addition of sulfamethoxazole to the polymer matrix. The bands at 1725 and 1145 cm^−1^ for the MIP samples are visible, which are of significantly higher intensity compared to the same bands of the corresponding NIP and washed samples. This is precisely the evidence that after washing all the prepared MIPs, the characteristic bands of the SMETOX in the polymer sorbent disappeared. This indicates that washing was complete.

The morphology and microstructure of the prepared MAA-based polymers were investigated by scanning electron microscopy.

**Figure 6:**
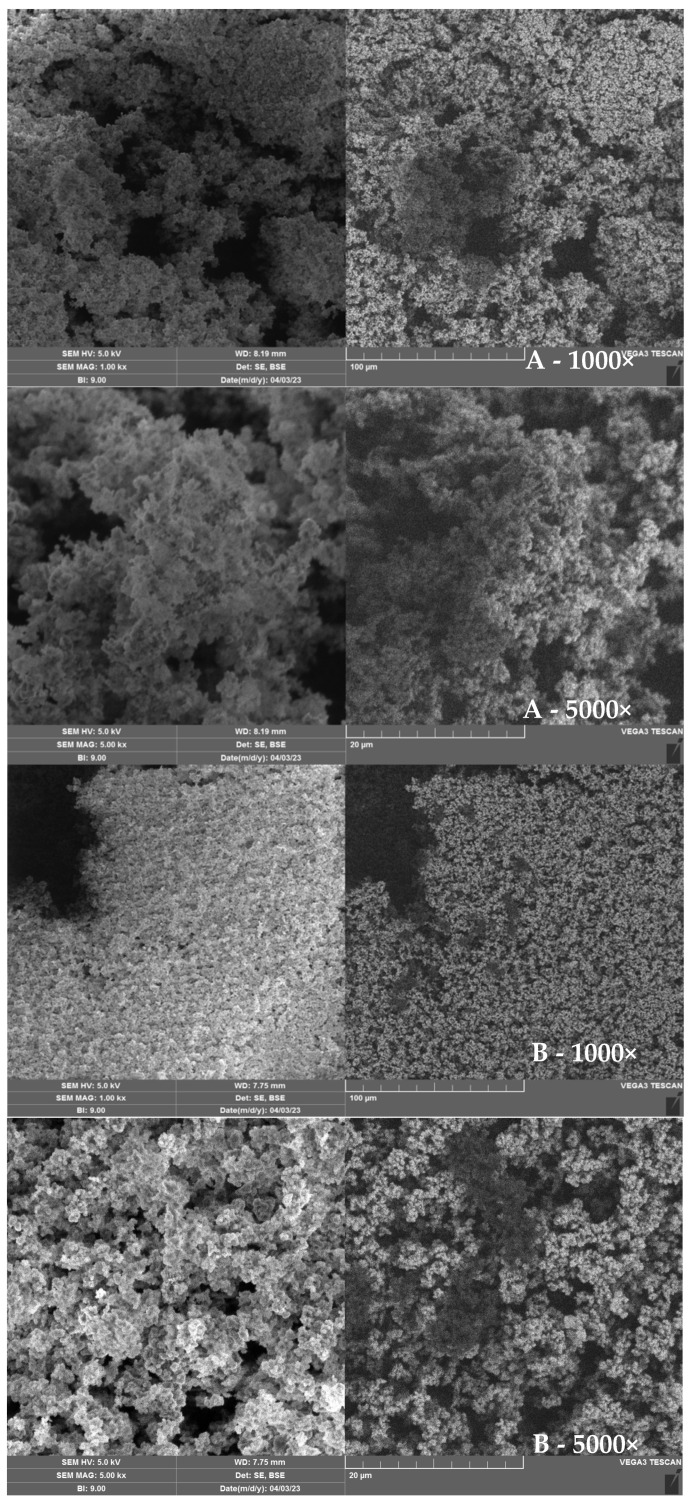
SEM images for MIP_MAA_ (**A**) and NIP_MAA_ (**B**) with 1000× and 5000× magnification.

SEM images of the prepared MIP-MAA and NIP-MAA polymers for solid phase extraction are shown in Figure 6.

Based on the SEM images, a network of irregular shapes with a large surface area is seen. Both samples showed a similar structure. Furthermore, it can be observed that the sulfamethoxazole-imprinted polymer has a rougher surface corresponding to the increase in surface area, which obviously has larger pores compared to the polymer without the imprint. NIP-MAA polymer has a more compact surface with fewer pores. All this supports the presence of the imprinted sulfamethoxazole molecules [78]. The size of the pores is directly related to the adsorption capacity, mass transfer, and diffusion resistance because the newly formed pores can increase the sorption capacity, facilitate mass transfer, and reduce diffusion resistance. These results support the better retention of SMETOX with MIP as previously mentioned.

### 3.4. Optimization of MIP-SPE-HPLC-DAD Method

Since the initial objectives were achieved in the third attempt to produce MIP, i.e., a sulfamethoxazole-imprinted polymer was obtained that behaved satisfactorily in extraction experiments after the prior removal of the template; it was used in series with a commercial HLB sorbent. In one series connection, the commercial sorbent was the first; in the second series connection, it was the second. The results were compared to those obtained by the extraction on the Oasis HLB sorbent itself and are shown in Table 4.

Based on the results presented in Table 4, it is concluded that the optimal series between the commercial HLB sorbent and the prepared polymer sorbent MIP_MAA 3_ is the one in which the commercial sorbent was the first in the series. Such a result was to be expected, since the eleven tested pharmaceuticals first come into contact with the commercial sorbent during extraction, on which they are mostly effectively retained, and then, when they pass through the prepared MIP_MAA 3_, its active sites remain more available for sulfamethoxazole. In general, comparing the systems where solid-phase extraction was performed, the addition of MIP for SMETOX primarily increased the recovery of the target molecule, thereby confirming its selectivity and the purpose of its use.

### 3.5. Molecular Dynamic Analysis of Obtained Extraction Results

**Figure 7:**
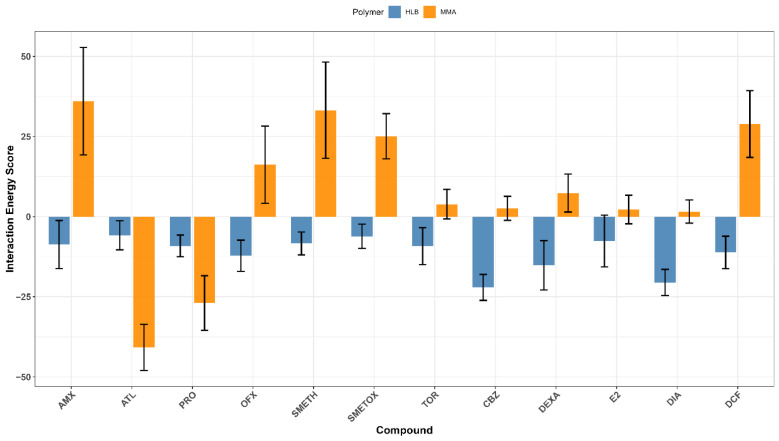
Interaction energy scores (in kcal/mol) of compounds with HLB and MAA polymers. Bar chart showing the interaction energies scores for various compounds with hydrophilic-lipophilic balance (HLB, blue) and methacrylic acid (MAA, orange) polymers. Scores were obtained with gmx_MMPBSA. Each bar represents the average interaction score, with standard deviation shown.

To evaluate whether experimental data could be accurately predicted using in silico methods, mirror systems replicating experimental setups were prepared to observe the interactions between post-polymerization polymers and selected pharmaceuticals. According to our methods, the systems were constructed following established protocols. Systematic testing across diverse polymer-pharmaceutical combinations revealed that a packing density of approximately 11–12 polymer atoms per nm^3^ provided the highest stability. This optimal configuration, consisting of roughly 2500 polymer atoms within a 6 × 6 × 6 nm simulation box, was chosen for further analysis. For systems containing HLB polymers, this density corresponded to eight polymer chains, each comprising five monomers. In contrast, systems with MAA polymers were composed of 20 polymer chains, each containing ten monomers. To further elucidate the interaction energy scores of the investigated compounds (monomers) with the hydrophilic-lipophilic balance and methacrylic acid polymers, we conducted an energy contribution analysis using molecular dynamics (MD) and the gmx_MMPBSA protocol [64]. This method provides end-state free energy calculations with GROMACS, factoring in van der Waals, polar solvation, and nonpolar solvation energies. The average energy contributions are visualized in Figure 7 and detailed in Table 5. This analysis allows us to understand the specific interactions driving the interactions between the polymers (HLB, MAA) and monomers, offering insights into potential optimization strategies for enhancing HLB/MAA-monomer interactions.

Resulting systems showed a wide range of interaction energies (kcal/mol, Figure 7). Due to limitations with estimating free binding energy with gmx_MMPBSA for these specific configurations, interaction energy was taken as a substitute.

All compounds exhibited favorable interaction scores with HLB; diazepam and dexamethasone had the strongest interaction. In contrast, only atenolol and procaine show affinity for MAA, with other compounds showing less favorable interactions. Notably, the docking scores for sulfamethoxazole and amoxicillin with MAA displayed significant variability, as reflected by larger standard deviation bars.

Based on the results presented in Table 5, the ATL-MAA complex demonstrates a favorable interaction score of −40.8 kcal/mol, primarily driven by strong non-polar interactions (−2830.0 kcal/mol). In contrast, the SMETOX-MAA complex shows unfavorable interaction, reflected by a positive total interaction score of 25.1 kcal/mol, indicating that the interaction is not spontaneous under the simulated conditions. The β-estradiol-HLB complex exhibits a favorable interaction with a ΔG_interaction_ of −7.6 kcal/mol, supported by significant van der Waals interactions. The PRO-HLB complex shows a relatively favorable interaction score of −9.1 kcal/mol, which is slightly less favorable than that of MAA, but still indicates spontaneous interaction. Similarly, the DCF-HLB and DEXA-HLB complexes yield very favorable interaction scores of −11.2 and −15.2 kcal/mol, respectively, suggesting strong interaction affinities characterized by excellent van der Waals interactions. Additionally, the DIA-HLB and CBZ-HLB complexes reflect highly favorable interactions, with a ΔG_interaction_ of −20.5 and −22.1 kcal/mol, respectively, where strong van der Waals interactions serve as the primary contributing factor. Overall, complexes involving HLB consistently exhibit more negative ΔG_interaction_ values compared to MAA, indicating enhanced interaction affinities. Notably, the non-polar contribution (ΔGGB (non-polar)) is often negative, underscoring the critical role of non-polar interactions in stabilizing these complexes.

### 3.6. Validation of MIP-SPE-HPLC-DAD Method and Comparison with SPE-HPLC-DAD Method

After using the prepared MIP_MAA 3_ polymer sorbent together with commercial Oasis HLB sorbent, the whole MIP-SPE-HPLC-DAD method was validated. To determine the performance characteristics of the validation, such as linearity, detection and quantification limits, and repeatability and reproducibility, dilutions of the standard solution of the pharmaceutical mixture were prepared.

The linearity of the method was tested for all eleven drugs in a concentration range from 0.0001 mg/L to 0.05 mg/L. A linear range was determined for all pharmaceuticals, and the results were analyzed using the linear regression method. LOD and LOQ were determined in the manner described above (Section 3.1) and range from 0.00025 to 0.001 mg/L for LOD and from 0.00025 to 0.001 mg/L for LOQ. Table S3 shows all LOD and LOQ values, the correlation coefficient (*R*^2^), and the dependence function. It can be seen that *R*^2^ is greater than 0.9932 for all drugs except diclofenac, β-estradiol, and dexamethasone, and the linearity of the method can be confirmed in the specified concentration range. In addition, the precision of the method was determined, expressed by repeatability and reproducibility, which are expressed as RSD. The repeatability of the method was tested for two concentrations of the pharmaceutical mixture in spring water: 1 and 2 mg/L. The recovery rate was calculated based on the data obtained by performing the experiment over three days.

Table S3 shows the RSD values for repeatability and reproducibility experiments for the listed pharmaceuticals in the mixture. The RSD values for repeatability do not exceed 4.99% for all pharmaceuticals, while for reproducibility, they do not exceed 10.62%.

When comparing all specific validation parameters determined for both methods (SPE-HPLC-DAD and MIP-SPE-HPLC-DAD), it can be seen that the MIP-SPE-HPLC-DAD method is generally better, which was to be expected. Although there were no significant changes in the limit of quantification for most pharmaceuticals, the limit of detection was lower for atenolol, ofloxacin, and carbamazepine. The greatest change was obtained for sulfamethoxazole at both the limit of detection and the limit of quantification. Sulfamethoxazole can be determined with the new method in a significantly broader linear range than with the previous method. This means that the original and most important objective has become obsolete. The reproducibility is better for most pharmaceuticals, with the exception of procaine, while the reproducibility is only worse for sulfamethazine, dexamethasone, and β-estradiol. However, these are not significant changes, but the observed differences can be treated as experimental errors caused by multiple measurements.

**Figure 8:**
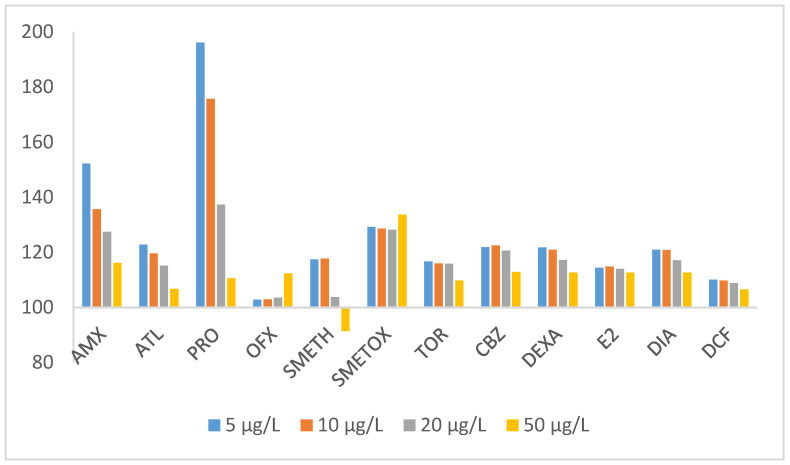
The matrix effect on MIP-SPE-HPLC-DAD method (initial pH of water = 8, elution solvent = methanol, elution volume = 2 × 2 mL, sample volume = 100 mL).

As already mentioned, the influence of the matrix on the analytes is particularly significant in pharmaceutical investigations and was determined in the same way as described in Section 2.5, and for the MIP-SPE-HPLC-DAD method with the sulfamethoxazole-imprinted polymer used. Figure 8 shows the influence of the matrix of 12 pharmaceuticals on their concentrations in water of 5, 10, 20, and 50 µg/L, using a polymer with a molecular imprint of sulfamethoxazole (MIP).

On the basis of Figure 8 and the comparison with Figure 5 for the case where no MIP was used, some differences but also similarities were identified. The matrix effect for the tested pharmaceutical mixture is still present, although it has changed due to the use of the MIP-SMETOX sorbent. The tendency of the matrix effect decreased in the case of SMETH such that it is only present in the MIP-SPE-HPLC-DAD method at the lowest concentrations, while it is no longer present at all at a concentration of 50 µg/L. In the case of ATL, it remained the same, only the range widened a little, while the biggest shift for the better occurred with OFX, so that the matrix effect almost disappeared when the MIP-SMETOX sorbent was used. MIP-SMETOX as a sorbent is not suitable for PRO, as in its presence, the matrix effect for PRO changed strongly for the worse (196–110%); this is also the largest matrix effect. It was expected that the reaction of SMETOX would increase in the presence of its specific sorbent, although the tendency of this influence is independent of the SMETOX concentration detected in the water. The pronounced positive matrix effect for PRO is most likely related to its strong affinity for monomer methacrylic acid (MAA) and residual SMETOX sites within the SMETOX–MIP. Structural similarities with the template molecule and observed non-specific interactions led to the occupation of imprinted cavities by procaine. TOR, CBZ, DEXA, E2, and DIA confirm that the MIP sorbent used is not the best solution for all analyzed pharmaceuticals, as for them, a slight matrix effect of the water sample occurs in the presence of MIP-SMETOX (signal increase of up to 10%). It is perhaps possible to assume that the structure of the SMETOX imprint does not completely match the structures of the analyzed pharmaceuticals, as some parts of their molecules may remain trapped or encapsulated in the sorbent. In general, however, we can say that by using a specific sorbent (in this case, MIP-SMETOX), we have made some progress compared to the situation where this specific polymer was not used. This is certainly confirmed by AMX, a pharmaceutical that constantly “escaped” in the previous SPE-HPLC-DAD method and did not remain bound to the sorbent, which is why we could not extract it from the water. By applying the MIP-SPE-HPLC-DAD method, we have finally succeeded.

### 3.7. Application of Both Developed Methods to Wastewater Samples

To demonstrate the applicability of the developed methods, two samples of hospital wastewater were analyzed (Table 6 and Table 7). The difference between the analyzed wastewater samples is the sampling location in the water treatment plant. The extraction was performed in three steps according to the standard addition calibration method. The first series was wastewater without the addition of a standard solution, while the other two were spiked with a pharmaceutical mixture at concentrations of 0.5 mg/L and 1 mg/L. Chromatograms for the non-spiked and spiked wastewater samples are attached as Figure S2 in Supplementary Materials.

Looking at the results of the performed analyses presented in Table 6 and Table 7, a fairly good match is visible. Of the 11 pharmaceuticals tested, the presence of at least 9 of them was confirmed, depending on the analyzed sample. The biggest difference is observed in the quantification of sulfamethoxazole, which is, of course, in line with expectations. However, MIP-SPE-HPLC-DAD is a more sensitive method for sulfamethoxazole because the sorbent was prepared with its print. The performed analyses confirm the need to develop analytical methods as well as their improvement, given that various water sources, especially wastewater, are also contaminated with pharmaceuticals. In particular, the focus is on low concentration detection levels and, accordingly, on the development of more sensitive methods that will allow the quantification of target substances such as SMETOX in this work through the preparation and application of MIP sorbents. In addition to the MIP-SMETOX-HPLC-DAD method described above, Table 8 lists other studies previously published for the application of different MIPs for the analysis of pharmaceuticals from various water matrices.

Table 8 shows that the results obtained in this paper (LOD or LOQ) are not even slightly behind the results obtained in previous publications. The results are even better, especially considering that HPLC-DAD was used in this paper as opposed to LC-MS/MS, which was used in some of the previous publications. This should be especially highlighted when comparing studies that used the same template, confirming the constant necessity for method development, including MIPs, which improve method sensitivity for the template molecule, and can be expanded for other analytes from the sample matrix.

### 3.8. Analysis of the MIP Regeneration Performance

**Figure 9:**
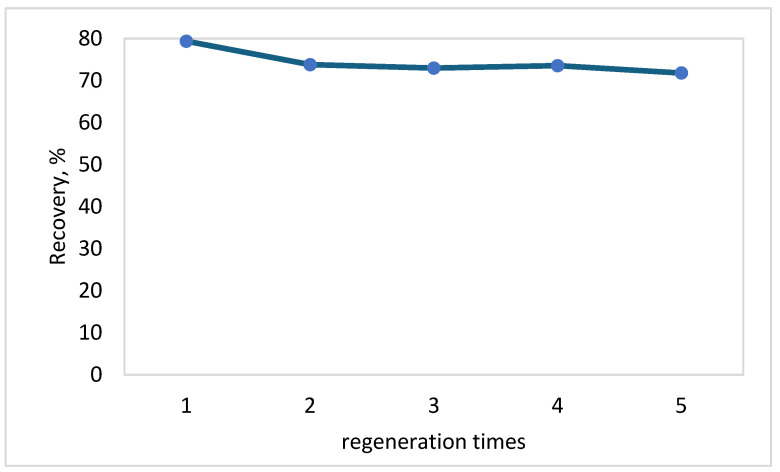
Regeneration efficiency of MIP-SMETOX.

**Figure 10:**
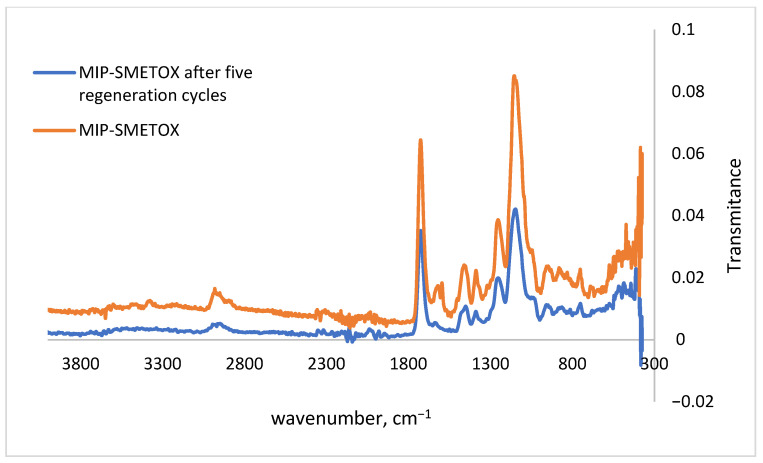
FTIR spectra of MIPs-SMETOX.

During the five repeated extraction cycles (Figure 9), the extraction efficiency of MIP-SMETOX decreased slightly, characterizing the polymer as an efficient and stable material that can be reused. The FTIR spectra (Figure 10) of the sorbent used five times in a row, compared with the MIP powder recorded after washing the template, show the same band positions, indicating the chemical stability of the polymer matrix. The slight drop in peak intensity and extraction efficiency, therefore, results from possible physical changes to the material surface and the presence of sorption residuals.

## 4. Conclusions

Solid-phase extraction is an effective technique frequently used for concentration and separation of molecules from different matrices, allowing the possibility to analyze different organic pollutants present in the environment at small concentration ranges. Accordingly, this work includes the development of the SPE method coupled with HPLC-DAD for the investigation of eleven pharmaceuticals from the water matrix. In parallel, a polymer sorbent with imprinted sulfamethoxazole was synthesized using MAA and HEMA as monomer units and EDGMA as cross-linker, after which the new material, MIP-MAA, was successfully applied for extracting sulfamethoxazole itself and other pharmaceuticals in the mixture. Results obtained with the first method, which included the use of a commercial HLB sorbent, achieved recoveries between 75 and 100%. In the next set of the SPE method, MIP material was involved in extraction, with commercial sorbent in two serial combinations, depending on the position of the sorbent in the cartridge. The best extraction for sulfamethoxazole and the rest of the mixture was achieved when the commercial sorbent was the first in the series. Both validated methods showed applicability on more complex water matrices, like wastewater, where the presence of this kind of pollutant is expected and even confirmed for some. Also, this study demonstrates the emphasized applicability of MIP, not only toward the template molecule, but it can also effectively react with other molecules, increasing the cross-selectivity factor from different water matrices. Matrix effect decreased for sulfamethoxazole, while the use of the MIP sorbent caused an enhancement of the peak for procaine. MD analysis was performed to support the obtained extraction results in terms of complementarity between in silico methods and experimental data.

## Figures and Tables

**Table 1:** Gradient elution for pharmaceuticals separation.

*t*, min	Eluent A, %	Eluent B, %
0.00	98.0	2.0
2.00	98.0	2.0
22.00	60.0	40.0
25.00	50.0	50.0
27.00	50.0	50.0
32.00	10.0	90.0
35.00	2.0	98.0
36.00	2.0	98.0
36.01	98.0	2.0
40.00	98.0	2.0

**Table 2:** Efficiency of sulfamethoxazole extraction from water using the MIP and NIP polymer sorbents (two functional monomers: MAA and HEMA).

Prepared MIPs	Extraction Efficiency, % (*n* = 3)
MIP_HEMA_	6.12 ± 3.59
NIP_HEMA_	1.83 ± 2.67
MIP_MAA 1_	7.19 ± 5.66
NIP_MAA 1_	2.56 ± 7.31

**Table 3:** Efficiency of sulfamethoxazole extraction from water using the 6 mL (MIP_MAA 2_) or 12 mL (MIP_MAA 3_) of acetonitrile in the preparation procedure of MIP and NIP polymer sorbents.

Prepared MIPs	Extraction Efficiency, % (*n* = 3)
MIP_MAA 2_	39.48 ± 11.02
NIP_MAA 2_	2.91 ± 5.42
MIP_MAA 3_	79.37 ± 9.25
NIP_MAA 3_	2.82 ± 4.25

**Table 4:** Extraction efficiency of sulfamethoxazole and other pharmaceuticals in the mixture of water using serial bonding of the best prepared MIP sorbent and the commercial HLB sorbent (MIP_MAA 3_ and Oasis_HLB_) in two different combinations (depending on which is the first in the series).

Pharmaceuticals	Extraction Efficiency, % (*n* = 3)		
	Oasis_HLB_	MIP_MAA 3_—Oasis_HLB_	Oasis_HLB_—MIP_MAA 3_
ATL	92.57 ± 2.37	73.81 ± 10.62	85.33 ± 4.72
PRO	89.12 ± 0.20	65.56 ± 8.61	86.75 ± 5.32
OFX	86.53 ± 7.24	92.49 ± 11.93	77.78 ± 2.57
SMETH	75.23 ± 3.47	109.40 ± 5.14	90.44 ± 0.52
SMETOX	78.01 ± 4.92	100.44 ± 8.41	102.49 ± 2.40
TOR	96.76 ± 0.92	101.05 ± 3.60	95.23 ± 2.93
CBZ	98.75 ± 1.60	80.83 ± 9.62	96.10 ± 4.58
DEXA	99.96 ± 1.00	105.55 ± 7.12	103.76 ± 7.82
E2	98.09 ± 0.83	76.48 ± 11.06	97.25 ± 6.33
DIA	96.99 ± 1.48	71.54 ± 10.77	91.79 ± 1.20
DCF	91.89 ± 1.73	99.17 ± 5.55	95.49 ± 3.44

**Table 5:** Important thermodynamic parameters during 100 ns MD simulation generated with gmx_MMPBSA protocol.

Complex	ΔG_vdW_ (kcal/mol)	ΔG_GB (polar)_ (kcal/mol)	ΔG_GB (non-polar)_ (kcal/mol)	ΔG_interaction_ (kcal/mol)
AMX-MAA	−6.1	−2245.3	2281.3	36.0
AMX-HLB	−11.5	2.4	−11.1	−8.7
ATL-MAA	−2.0	2789.2	−2830.0	−40.8
ATL-HLB	−7.9	3.6	−9.4	−5.8
PRO-MAA	−2.6	2280.4	−2307.3	−26.9
PRO-HLB	−12.3	4.8	−13.9	−9.1
OFX-MAA	−10.0	72.2	−56.0	16.2
OFX-HLB	−17.0	6.9	−19.0	−12.2
SMETH-MAA	−1.9	−2055.9	2089.1	33.2
SMETH-HLB	−9.2	0.7	−9.1	−8.4
SMETOX-MAA	−0.8	−1868.5	1893.6	25.1
SMETOX-HLB	−9.2	0.7	−9.1	−8.4
TOR-MAA	−3.2	−13.6	17.5	3.9
TOR-HLB	−10.3	1.5	−10.7	−9.2
CBZ-MAA	−1.4	−3.2	5.7	2.6
CBZ-HLB	−26.7	5.6	−27.6	−22.1
DEXA-MAA	−12.2	84.4	−77.1	7.4
DEXA-HLB	−17.0	2.1	−17.3	−15.2
E2-MAA	−2.0	−10.6	12.8	2.2
E2-HLB	−7.6	0.1	−7.7	−7.6
DIA-MAA	−1.4	8.5	−6.9	1.6
DIA-HLB	−23.4	3.5	−24.0	−20.5
DCF-MAA	−1.0	−2018.1	2047.0	28.9
DCF-HLB	−12.3	0.8	−12.0	−11.2

**Table 6:** Application of SPE-HPLC-DAD method for detection of pharmaceuticals in wastewaters.

Pharmaceuticals	LOD, µg/L	LOQ, µg/L	WW1	WW2
ATL	0.5	1.0	<LOQ	<LOQ
PRO	0.25	0.5	<LOQ	<LOQ
OFX	0.25	0.5	4.95	1.61
SMETH	0.25	0.5	LOD	<LOQ
SMETOX	0.5	1.0	<LOD	<LOQ
TOR	0.1	0.25	<LOD	0.46
CBZ	0.1	0.25	5.12	2.11
DEXA	0.25	0.5	n.d.	<LOQ
E2	0.25	0.5	<LOD	n.d.
DIA	0.1	0.25	0.34	0.35
DCF	0.1	0.25	n.d.	1.27

n.d.—not detected.

**Table 7:** Application of MIP-SPE-HPLC-DAD method for detection of pharmaceuticals in wastewaters.

Pharmaceuticals	LOD, µg/L	LOQ, µg/L	WW1	WW2
ATL	0.25	1.0	<LOQ	<LOQ
PRO	0.25	0.5	<LOQ	<LOQ
OFX	0.1	0.25	5.05	1.65
SMETH	0.25	0.5	LOD	<LOQ
SMETOX	0.1	0.25	0.35	0.64
TOR	0.25	0.5	<LOD	<LOQ
CBZ	0.25	0.5	5.21	2.01
DEXA	0.25	0.5	n.d.	<LOQ
E2	0.25	0.5	<LOD	n.d.
DIA	0.1	0.25	0.36	0.36
DCF	0.1	0.25	n.d.	1.32

n.d.—not detected.

**Table 8:** Comparison table of previously published MIP-SPE methods with this work.

Template Molecule/Analyte	SPE Parameters	Method	Reference
diclofenac	35 mg of MIP, 1 L of wastewater, elution with 2 mL of methanol/acetic acid (9:1, *v*:*v*)	LC–MS/MS LOD—not reported	[81]
ketoprofen	14 mg of MIP, 50 mL of wastewater, pH 5, elution with 1 mL methanol	LC-UV LOD—0.23 μg/L	[82]
non-steroidal anti-inflammatory drugs	150 mg of MIP, 250 mL of wastewater, pH 3, elution with 5 mL 1% CH_3_COOH in MeOH/acetone (80:20)	LC-UV, LC-MS/MS	[83]
venlafaxine (MIP), carbamazepine, etilefrine, methocarbamol, nevirapine, venlafaxine	50 mg of MIP, 60 mL of dam water sample, pH 6, elution with 1% (*v*/*v*) formic acid in methanol	LC-MS LOQ, µg/L: VFX 1, CBZ 0.13, ETF 0.12, MTC 1.03, NVP 3.81	[38]
oxazepam (MIP), diazepam, temazepam, nordiazepam	50 mg of MIP, 0.5 mL of urine, 2 × 0.5 mL of acetic acid–methanol (20:80, *v*/*v*)	HPLC-DAD LOQ, µg/L: OZ 53.5, TZ 63.9, NZ 44.5, DZP 69.3	[36]
sulfamethoxazole magnetic MIP	6 g/L MIP, sorption of SMX 10 min and elution with acetonitrile (10 min of stirring)	UV/Vis LOD—0.59 µM, LOQ—1.77 µM	[84]
sulfamethoxazole	MIP-PMME method, 5 mL of milk, pH 2, 0.1 mL acetonitrile for elution	HPLC-DAD LOD—1 µg/L	[85]
sulfamethoxazole (MIP) + 11 pharmaceuticals	60 mg of Oasis HLB + 60 mg of MIP, 100 mL of wastewater, pH 8, elution with 2 mL methanol	HPLC-DAD LOQ—Table 7	This paper

## Data Availability

Data will be made available upon request.

## Supplementary Materials

The following supporting information can be downloaded at https://www.mdpi.com/article/10.3390/polym17233203/s1: Figure S1: chromatogram obtained from the standard solution of the pharmaceutical mixture used; Figure S2: chromatogram obtained for non-spiked (A) and spiked (B) wastewater sample with standard solution of pharmaceutical mixture; Table S1: information of pharmaceuticals used; Table S2: results of quantitative determination of pharmaceuticals in water by SPE-HPLC-DAD; Table S3: results of quantitative determination of pharmaceuticals in water by MIP-SPE-HPLC-DAD.
