# Supplementary material for: Preparation and Application of Sulfamethoxazole-Imprinted Polymer on Solid-Phase Extraction of Pharmaceuticals from Water
Source: Polymers (Basel). 2025 Nov 30;17(23):3203. doi: 10.3390/polym17233203 (PMC12694196; doi:10.3390/polym17233203)
Supplement: Supplementary file 1 [file polymers-17-03203-s001.zip › polymers-3985167-supplementary.pdf]

Supplementary Materials

Preparation and application of sulfamethoxazole- imprinted polymer on solid-phase extraction of pharmaceuticals from water

Kristina Tolić Čop[1], Stjepan Jozinović[1], David Visentin[2], Dejan Milenković[3], Petra Vukovinski[1], Ramona Petko[1], Robert Vianello[4], Dragana Mutavdžić Pavlović[1]*

[1]Department of Analytical Chemistry, Faculty of Chemical Engineering and Technology, University of Zagreb, Trg Marka Marulića 19, 10000 Zagreb, Croatia

[2]Laboratory for the Computational Design and Synthesis of Functional Materials, Ruđer Bošković Institute, Bijenička cesta 54, 10000 Zagreb, Croatia

[3]Department of Molecular and Systemic Biomedicine, Faculty of Biotechnology and Drug Development, University of Rijeka, Ul. Radmile Matejčić 2, 51000, Rijeka, Croatia

[4]Department of Science Institute for Information Technologies, University of Kragujevac, Jovana Cvijića bb34000, Kragujevac, Serbia

List of figures:

Figure S1. Chromatogram obtained from the standard solution of the pharmaceutical mixture used

Figure S2. Chromatogram obtained for non-spiked (A) and spiked (B) wastewater sample with standard solution of pharmaceutical mixture

List of tables:

Table S1. Information of pharmaceuticals used

Table S2. Results of quantitative determination of pharmaceuticals in water by SPE-HPLC-DAD

Table S3. Results of quantitative determination of pharmaceuticals in water by MIP-SPE-HPLC-DAD

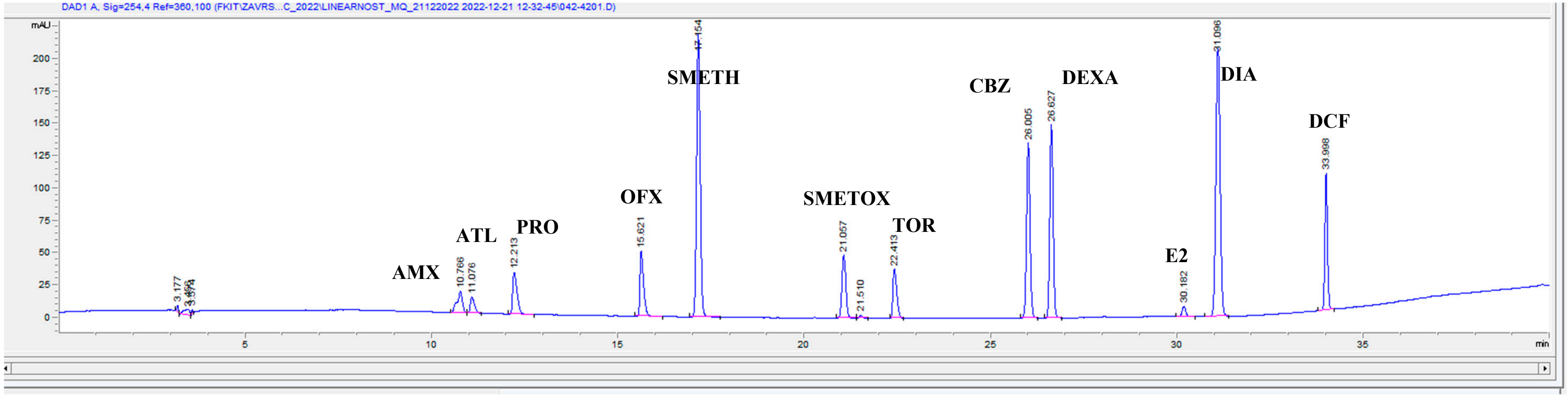

Figure S1. Chromatogram obtained from the standard solution of the pharmaceutical mixture used

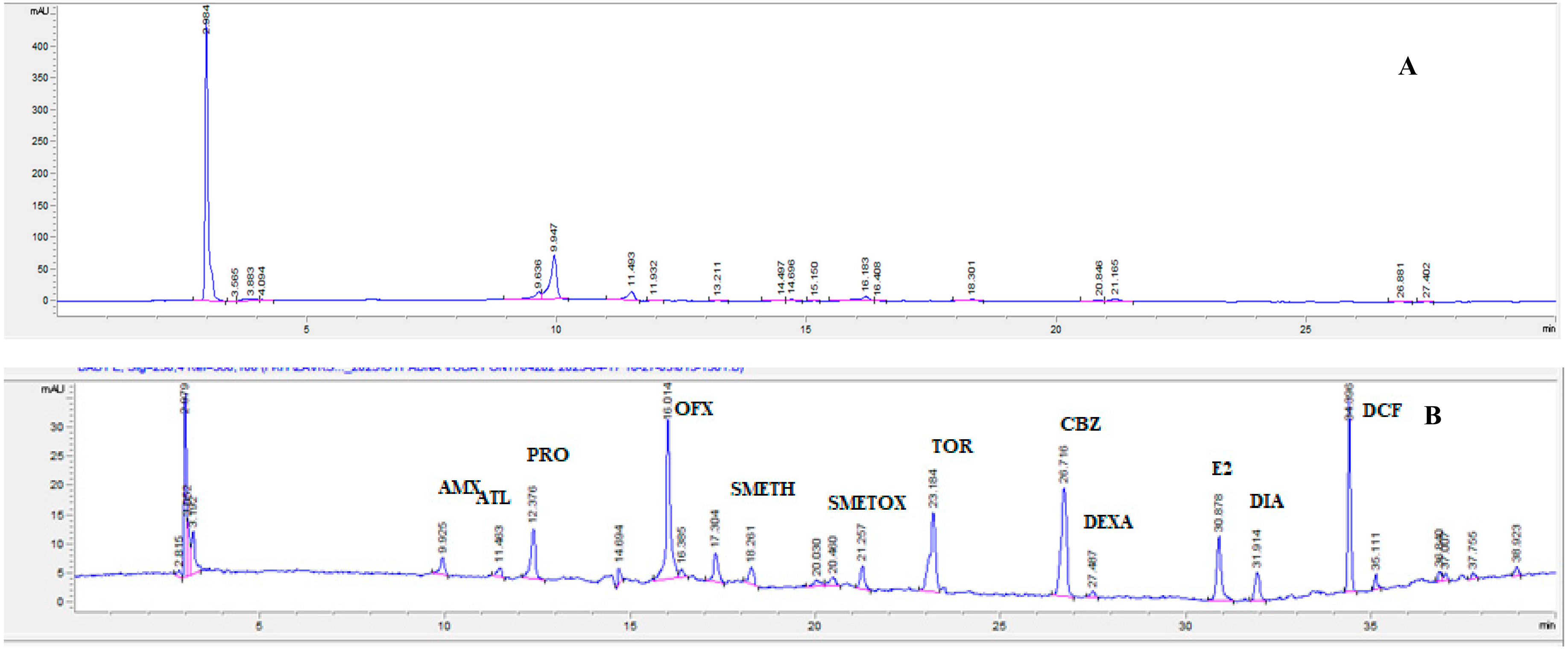

Figure S2. Chromatogram obtained for non-spiked (A) and spiked (B) wastewater sample with standard solution of pharmaceutical mixture

Table S1. Information of pharmaceuticals used

Chemical structure	Empirical formula	CAS	M_w	pK_a	log K_{ow}	Absorption maximum, nm	Supplier	Class
Amoxicilin 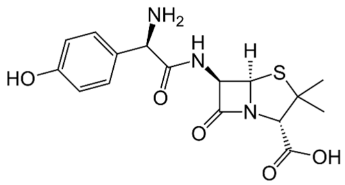	$C_{16}H_{19}N_3O_5S$	26787-78-0	365.4	3.2; 11.7	0.87	230	Genera d.o.o.	penicillin
Atenolol 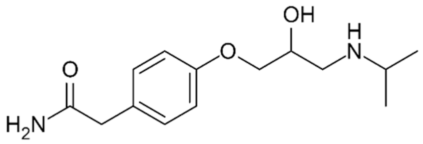	$C_{14}H_{22}N_2O_3$	29122-68-7	266.34	9.16	0.16	230	Acros Organics	β-blocker
Dexamethasone 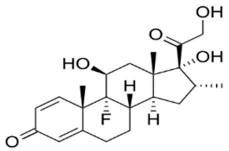	$C_{22}H_{29}FO_5$	50-02-2	392.5	1.18; 3.4	1.83	240	Acros Organics	corticosteroid
Diazepam 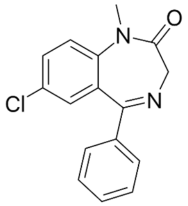	$C_{16}H_{13}ClN_2O$	439-14-5	284.74	3.4	2.82	230	JGL d.d.	anxiolytic
Diclofenac	$C_{14}H_{11}Cl_2NO_2$	15307-86-5	296.1	4.15	4.51	275	Sigma Life Science	nonsteroidal anti-inflammatory drug

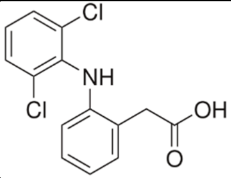								
Carbamazepine 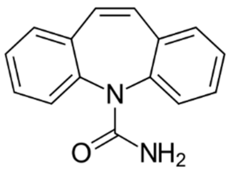	$C_{15}H_{12}N_2O$	298-46-4	236.27	15.96; -3.8	2.77	230	Sigma-Aldrich	antiepileptic
Ofloxacin 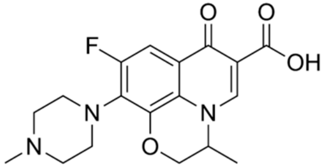	$C_{18}H_{20}FN_3O_4$	82419-36-1	361.4	8.31	-0.39	290	Acros Organics.	quinolone antibiotics
Procaine 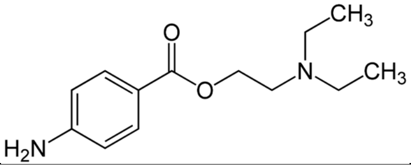	$C_{13}H_{20}N_2O_2$	59-46-1	236.31	8.05	1.92	290	Sigma Aldrich	local anesthetic
Sulfamethazine 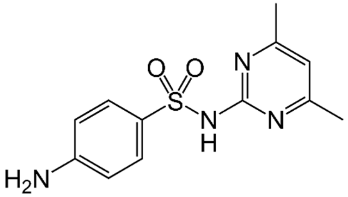	$C_{12}H_{14}N_4O_2S$	57-68-1	278.33	2.65; 7.65	0.89	275	Genera d.o.o	sulfonamide antibiotic
Sulfamethoxazole	$C_{10}H_{11}N_3O_3S$	723-46-6	253.28	1.6; 5.7	0.89	275	Sigma-Aldrich	sulfonamide antibiotic

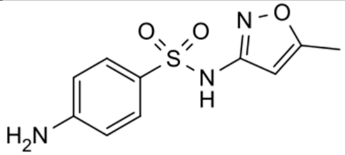								
Torasemide 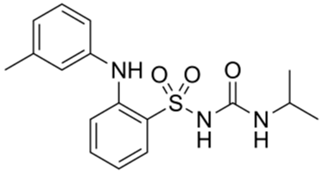	$C_{16}H_{20}N_4O_3S$	56211-40-6	348.4	7.1	3.36	290	Pliva d.o.o	diuretic
β-estradiol 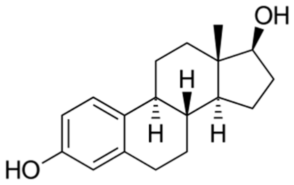	$C_{18}H_{24}O_2$	50-28-2	272.4	10.46	4.01	230	Acros Organics	hormone replacement therapy

Table S2. Results of quantitative determination of pharmaceuticals in water by SPE-HPLC-DAD

**Pharmaceutical**	**Linearity range, mg/L**	R^2	**LOD, mg/L**	**LOQ, mg/L**	**Repeatability, RSD (%) (n =5)**	**Reproducibility, RSD (%) (n =15)**
AMX	/	/	/	/	/	/
ATL	0.001 - 0.05	0.9984	0.0005	0.001	6.16	14.29
PRO	0.0005 – 0.05	0.9874	0.00025	0.0005	3.84	13.28
OFX	0.0005 – 0.05	0.9990	0.00025	0.0005	7.00	10.50
SMETH	0.0005 – 0.05	0.9923	0.00025	0.0005	4.65	7.04
SMETOX	0.001 – 0.05	0.9953	0.0005	0.001	9.39	11.61
TOR	0.00025 – 0.05	0.9820	0.0001	0.00025	3.65	10.65
CBZ	0.00025 – 0.05	0.9884	0.0001	0.00025	3.70	13.96
DEXA	0.0005 – 0.05	0.9894	0.00025	0.0005	3.74	4.94
E2	0.0005 – 0.05	0.9774	0.00025	0.0005	3.96	7.77
DIA	0.00025 – 0.05	0.9868	0.0001	0.00025	3.85	10.19
DCF	0.00025 – 0.05	0.9889	0.0001	0.00025	3.96	10.07

Table S3. Results of quantitative determination of pharmaceuticals in water by MIP-SPE-HPLC-DAD

Pharmaceutical	Linearity range, mg/L	R^2	LOD, mg/L	LOQ, mg/L	Repeatability, RSD (%) (n =5)	Reproducibility, RSD (%) (n =15)
AMX	/	/	/	/	/	/
ATL	0.001 - 0.05	0.9932	0.00025	0.001	2.15	8.55
PRO	0.0005 – 0.05	0.9992	0.00025	0.0005	4.99	9.42
OFX	0.00025 – 0.05	0.9961	0.0001	0.00025	2.98	9.38
SMETH	0.0005 – 0.05	0.9996	0.00025	0.0005	2.79	7.95
SMETOX	0.00025 – 0.05	0.9975	0.0001	0.00025	0.91	6.52
TOR	0.0005 – 0.05	0.9996	0.00025	0.0005	1.65	5.41
CBZ	0.0005 – 0.05	0.9971	0.00025	0.0005	0.78	6.81
DEXA	0.0005 – 0.05	0.9693	0.00025	0.0005	0.26	7.73
E2	0.0005 – 0.05	0.9512	0.00025	0.0005	1.90	10.62
DIA	0.00025 – 0.05	0.9980	0.0001	0.00025	3.08	3.62
DCF	0.00025 – 0.05	0.9853	0.0001	0.00025	2.30	4.69
